# Pelvic Organ Prolapse Quantification After Pessary Removal: The Use of Upright MRI in POP Research

**DOI:** 10.1007/s00192-025-06182-2

**Published:** 2025-06-04

**Authors:** Annemarie van der Steen, Frank F. J. Simonis, Anique T. M. Grob

**Affiliations:** 1 Multi Modality Medical Imaging (M3I), TechMed Centre, University of Twente, Drienerlolaan 5, 7522 NB Enschede, The Netherlands; 2 Department of Gynecology, Ziekenhuisgroep Twente, Hengelo/Almelo, The Netherlands; 3 Magnetic Detection and Imaging (MD&I), TechMed Centre, University of Twente, Enschede, The Netherlands

**Keywords:** Magnetic resonance imaging, Pelvic inclination correction system, Pelvic organ prolapse, Pelvic organ quantification system, Pessary treatment, Upright

## Abstract

**Introduction:**

When pelvic organ prolapse (POP) patients change from pessary to surgical treatment, new POP quantification is often necessary. However, the time to maximal POP recurrence after pessary removal is unclear. This video-article illustrates the variation over time of POP extent after pessary removal.

**Methods:**

Upright MRI scans of 12 POP patients were used to measure the distances from the lowest points of bladder and cervix to the Pelvic Inclination Correction System (PICS)) line, with the pessary in situ, immediately, 4 and 8 h after pessary removal. Statistical differences between time points were determined.

**Results:**

The bladder descended immediately after pessary removal from a median of 0.1 cm above to 1.8 cm below the PICS line. In 58% of patients, the bladder then remained stable; in 33%, the bladder further descended up to 5.4 cm after 4 to 8 h. The cervix descended immediately after pessary removal from a median of 3.1 cm to 1.9 cm above the PICS line. In 17% of patients, a late cervix descent after 4–8 h was seen.

**Conclusion:**

POP quantification should be delayed at least 8 h after pessary removal to prevent underestimation of POP extent.

**Supplementary Information:**

The online version contains supplementary material available at 10.1007/s00192-025-06182-2.

## Introduction

Pessaries are for many POP patients the first line of treatment to alleviate their symptoms [1, 2]. Some patients, however, switch to surgical treatment because of failure of pessary therapy or side effects [3]. Especially after using a pessary for a longer time period, these patients need a new physical examination (Pelvic Organ Prolapse Quantification (POP-Q)) to determine the current extent of POP to allow for careful surgical planning. Currently, it is unknown whether the pelvic organs descend to their maximum extent directly after removal of the pessary, or if this descent is delayed. In the latter case, the POP-Q examination should not be performed immediately after removing the pessary. Because earlier research showed that supine examination with Valsalva underestimates the extent of POP [4], to accurately study the timing and amount of POP recurrence after pessary removal, we consider upright MRI a suitable method. Therefore, the aim of this video-article is twofold, showing the methodology that is used for upright MRI measurements in POP patients, and to show the variation in POP recurrence after pessary removal.

## Method

In this prospective study, upright MRI scans of 12 POP patients who were wearing their pessaries at least 3 months were used. All patients had at least stage 2 POP of the anterior and/or apical compartment and were 18 years or older. They were excluded if they were not able to stand for 20 min without assistance, were unable to refrain from lying down during the day, were not able to pass the MR safety checklist, or had a jeans size ≥52 (EU) or 22 (US) because of the limited coil circumference. The study was approved by the medical ethics committee and registered as NL74061.091.20. All patients were recruited at the outpatient clinic of our gynecology department and gave written informed consent. They had removed their pessary just before the scan. In addition, upright scans of the same 12 women with the pessary in situ were used. Scans after pessary removal were performed in the morning (8–10 am), midday (12 am–2 pm), and afternoon (4–6 pm). The exact methodology of MRI scanning and annotation of the landmarks was described before by Van der Steen et al. [5]. The Wilcoxon signed-rank test was used to evaluate statistically significant differences (*p* <.05) in bladder and cervix heights between the morning and midday/afternoon scans.

## Results

After initial inclusion, three patients were excluded from analysis since they removed their pessaries themselves the evening before the scan, leaving 12 patients for analysis. Table 1 shows the baseline characteristics of the population. POP-Q measurements with Valsalva, performed prior to pessary placement, were extracted from the electronic patient file. Patients were wearing their pessaries from 4 months up to 10 years.

**Table 1:** Baseline characteristics of the study population (*n* = 12)

Age (years)		68.0±6.4
BMI^a^		27.0 (22–38)
Parity^a^		2 (0–3)
POP-Q stage bladder^b^	Stage 1	1 (10%)
	Stage 2	6 (60%)
	Stage 3	3 (30%)
POP-Q stage uterus^b^	Stage 0	3 (30%)
	Stage 1	4 (40%)
	Stage 2	2 (20%)
	Stage 3	1 (10%)

Data presented as mean ± SD, median (range)^a^, or number of patients (percentage)^b^. *n* number of patients, *BMI* body mass index, *POP-Q* Pelvic Organ Prolapse Quantification. POP-Q measurements were only known in 10 of 12 patients

### Bladder

With the pessary in situ, the lowest point of the bladder was measured at a median (min, max) of −0.1 cm (−0.8; 1.5) above the PICS line. Immediately after pessary removal, the lowest point of the bladder dropped on average 1.9 cm (*p* =.002) with a measured median (min, max) of 1.8 cm (−0.4; 4.4) below the PICS line. The median difference in bladder height as compared to the morning scans without pessary was 0.1 (−1.2; 2.7) cm after 4 h (midday) and 0.3 (−1.2; 5.4) cm after 8 h (afternoon); the latter being statistically significant (*p* =.023) (Table 2).

**Table 2:** Median differences of bladder and cervix height between morning, midday, and afternoon in 12 patients

	Bladder			Cervix		
	With vs without pessary	Morning vs midday	Morning vs afternoon	With vs without pessary	Morning vs midday	Morning vs afternoon
Median difference (min, max)	1.70 (0.35; 4.50) cm	0.11 (−1.17; 2.74) cm	0.32 (−1.23; 5.38) cm	1.07 (−0.19; 3.72) cm	0.36 (−1.00; 1.15) cm	0.39 (−0.57; 1.97) cm
*p* value (Wilcoxon)	**.002**	.875	**.023**	**.003**	.239	.050

**Fig. 1:**
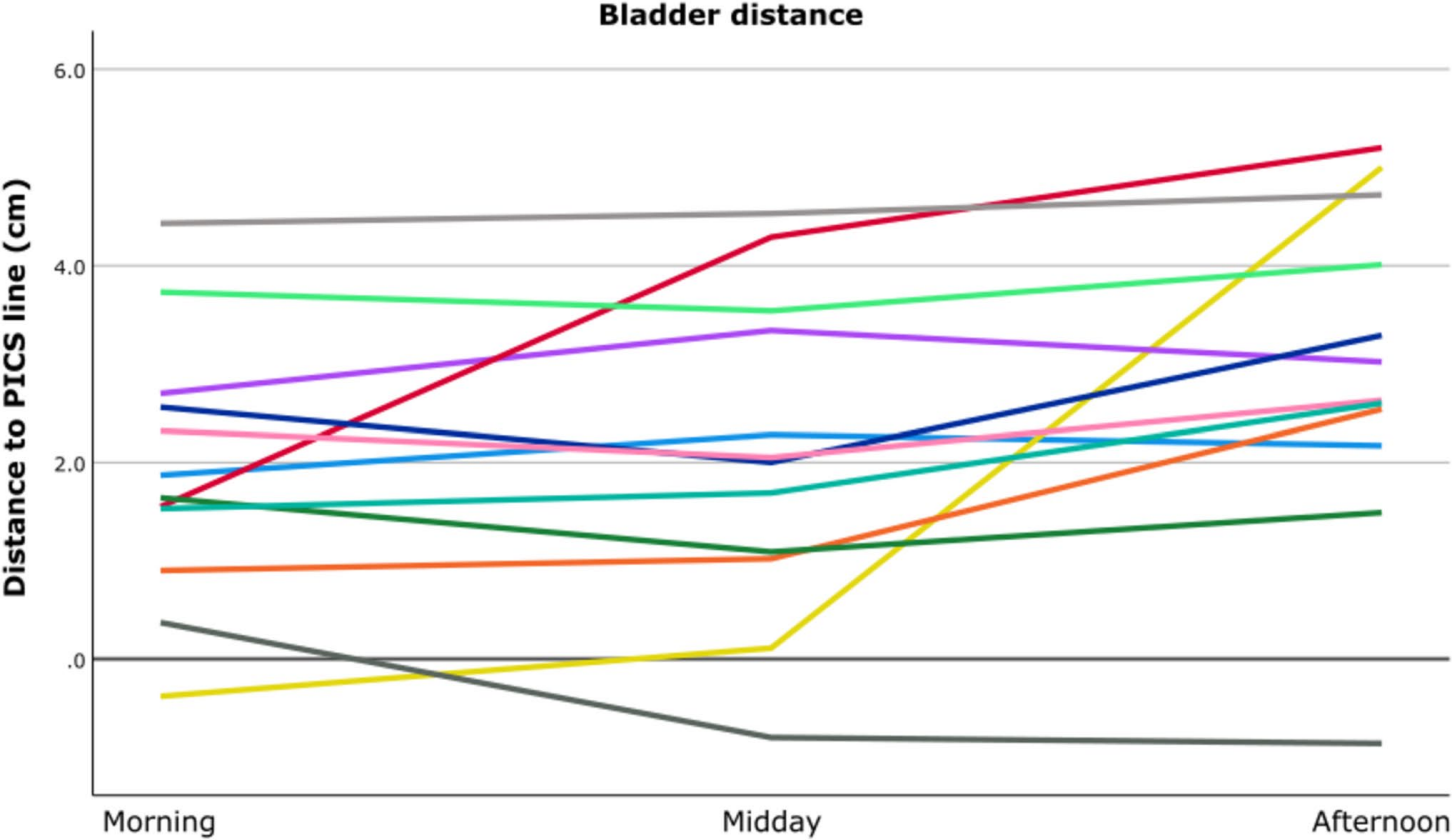
Distance of bladder height (cm) to PICS line for the individual patients at three moments of the day: morning, midday, and afternoon. Nearly all bladder heights are measured below the PICS line in the morning, some descend more during the day. *PICS line: pelvic inclination correction system line*

The variety in descent of the bladder after pessary removal is depicted in Fig. 1. In 58% of the patients, there is an immediate descent of the bladder after removal of the pessary, after which the bladder height is stable during the day. One patient shows an early delayed bladder descent of ≥1 cm between morning and midday, and three show a late delayed descent between midday and afternoon. Interestingly, in one patient after an initial drop of the bladder after pessary removal the prolapse decreases with 1.2 cm between morning and afternoon.

### Cervix

With the pessary in situ, the lowest point of the cervix was measured at a median (min, max) of −3.1 cm (−5.4; −1.2) above the PICS line. Immediately after pessary removal, the lowest point of the cervix dropped on average 1.2 cm (*p* =.003) with a measured median (min; max) of −1.9 cm (−4.3; 0.0). The median difference in cervix height was 0.4 (−1.0; 1.2) cm after 4 h (midday) and 0.4 (−0.6; 2.0) cm after 8 h (afternoon); both not statistically significant (Table 2).

**Fig. 2:**
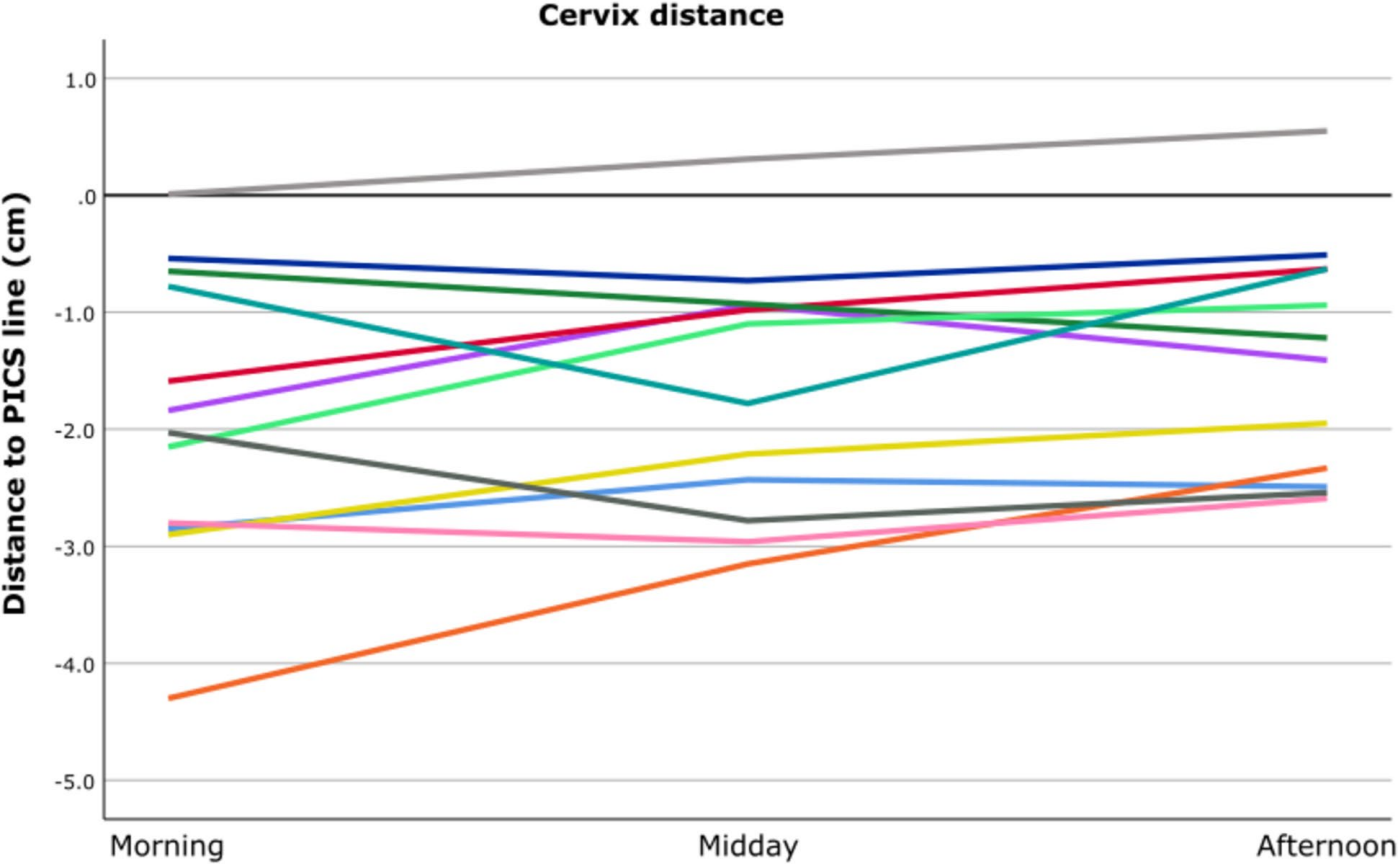
Distance of cervix height (cm) to PICS line for the individual patients at three moments of the day: morning, midday, and afternoon. Nearly all cervix heights are measured above the PICS line in the morning and stay above during the day. *PICS line: pelvic inclination correction system line*

The variety in cervix descent during the day is limited as is visualized in Fig. 2. In 25% of the patients, there is a limited descent of < 1 cm immediately after removal of the pessary, as well as during the day. In 58% of the patients, there is an immediate descent of 1 cm after which the cervix height is stable, and in 17%, there is a delayed descent between morning and midday.

## Discussion

This video shows the methodology of upright MRI scanning in POP patients and shows the variation that is seen in POP recurrence after pessary removal. In our population, a high inter-patient variation was found with regard to pelvic organ descent after pessary removal. In the majority of the patients (58%), we noticed an immediate descent of the bladder and uterus, without a clinically relevant further descent during the day. In 33% of the POP patients, there was a delayed descent which would have affected the POP evaluation, with individual differences of up to 5.4 cm. To the best of our knowledge, this is the first study that reports on the timing between pessary removal and POP evaluation. Previous studies did report on the daily variation of POP in patients without pessary using POP-Q [6, 7]. These studies did not find a difference between POP-Q measurements during the day. Additionally, in a previous study using upright MRI, we demonstrated that the natural descent of bladder and uterus during the day in a POP population was limited to a median of 0.3 cm with larger individual differences of up to 2 cm between morning and afternoon [5]. This indicates that the natural descent during the day is limited in most patients, and that the larger differences we see in the current study are most likely due to delayed effects of removal of the pessary.

We therefore conclude that POP-Q examination should be delayed at least 8 h after the removal of the pessary to ensure an accurate quantification of the prolapse.

## Supplementary Information

Below is the link to the electronic supplementary material.

Supplementary file1 (MP4 93487 KB)

## Data Availability

Data is available upon reasonable request.
